# Printing Composites with Salt Hydrate Phase Change Materials for Thermal Energy Storage

**DOI:** 10.1021/acsaenm.3c00324

**Published:** 2023-08-04

**Authors:** Sarah N. Lak, Chia-Min Hsieh, Luma AlMahbobi, Yifei Wang, Anirban Chakraborty, Choongho Yu, Emily B. Pentzer

**Affiliations:** † Department of Chemistry, Texas A&M University, College Station, Texas 77843, United States; ‡ Department of Materials Science and Engineering, Texas A&M University, College Station, Texas 77843, United States; § Department of Mechanical Engineering, Texas A&M University, College Station, Texas 77843, United States

**Keywords:** salt hydrate, phase change material, graphene oxide, encapsulation, 3D printing, direct ink writing, additive manufacturing, thermal energy storage, thermoregulation

## Abstract

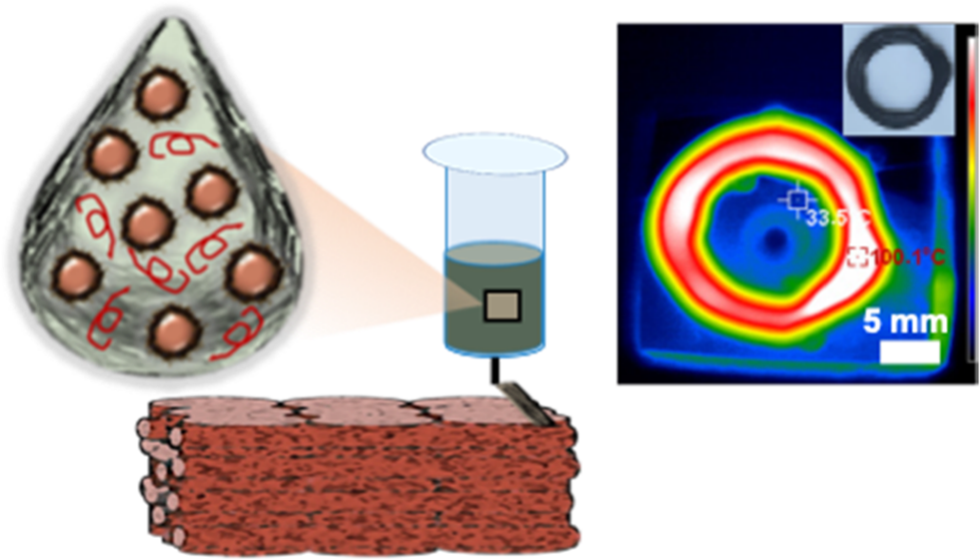

Salt hydrate phase change materials are important in advancing thermal energy storage technologies for the development of renewable energies. At present, their widespread use is limited by undesired undercooling and phase separation, as well as their tendency to corrode container materials. Herein, we report a direct ink writing (DIW) additive manufacturing technique to print noncorrosive salt hydrate composites with thoroughly integrated nucleating agents and thermally conductive additives. First, salt hydrate particles are prepared from nonaqueous Pickering emulsions and then employed as rheological modifiers to formulate thixotropic inks with polymer dispersions in toluene serving as the matrix. These inks are successfully printed at room temperature and cured by solvent evaporation under ambient conditions. The resulting printed and cured composites, containing up to 70 wt % of the salt hydrate, exhibit reliable thermal cyclability for 10 cycles and suppressed undercooling compared to the bulk salt hydrate. Remarkably, the composites consistently maintain their structural integrity and thermal performance throughout the entirety of both the melting and solidification processes. We demonstrate the versatility of this approach by utilizing two salt hydrates, magnesium nitrate hexahydrate (MNH, *T*_m_ = 89 °C) and zinc nitrate hexahydrate (ZNH, *T*_m_ = 36 °C), to achieve desired thermal characteristics across a wide range of temperatures. Further, we establish that the incorporation of carbon black in these inks enhances the thermal conductivity by at least 33%. This approach consolidates the strengths of additive manufacturing and salt hydrate phase change materials to harness customizable thermal properties, well suited for targeted thermal energy management applications.

## Introduction

As the global energy demand and anthropogenic greenhouse gas emissions continue to escalate, the widespread adoption of renewable energy resources has become increasingly crucial. The intermittent nature of many renewable energies remains one of the hurdles to be overcome so that they can be used in large-scale power generation technologies.^1^ This makes implementation of scalable, cost-effective, and sustainable energy storage solutions critical.^2^ An attractive and practical approach is the development and integration of thermal energy storage (TES) technologies that employ latent heat storage.^3^ Among TES media, phase change materials (PCMs) that absorb and release thermal energy by undergoing reversible solid–liquid phase transitions over well-defined temperatures are well suited for this purpose.^4^

As opposed to organic PCMs (e.g., paraffins, carbon-based fatty acids, and alcohols), salt hydrate PCMs have the advantages of high latent heat capacities, high volumetric energy densities, low cost, nonflammability, and reasonable thermal conductivities.^5^ Owing to their technological and economical sustainability, salt hydrate PCMs such as sodium sulfate decahydrate and sodium acetate trihydrate have been extensively explored in active TES-integrated equipment^6−8^ (such as HVAC systems) and TES systems for passive thermoregulation of buildings.^9−11^ However, the extensive use of salt hydrates is currently limited due to significant undercooling (where latent heat is not released at the melting point of the PCM),^12^ phase separation of components (which renders salt hydrates unstable to extended cyclability),^13^ and corrosiveness toward metals.^14^ Undercooling in salt hydrates has been suppressed by adding different nucleating agents.^15−17^ On the other hand, phase separation has been mitigated by the addition of thickeners such as carboxymethyl cellulose or polymers (e.g., polyvinyl alcohol).^18−20^

Encapsulation of salt hydrates within core–shell structures is an alternative approach that can be used to enhance thermophysical properties while preserving the energy storage capacities. Ideally, the shell would be mechanically robust and thermally conductive so as to accommodate volume changes during phase transitions and enhance the rate of heat transfer.^21,22^ Capsules of salt hydrates are usually prepared by *in situ* polymerization, interfacial polycondensation, solvent evaporation, and sol–gel methods.^23−30^ Many of these approaches do not use the desired salt hydrate composition and instead introduce extra water during the encapsulation process. Thus, such methods reduce the volumetric heat storage capacity of the salt hydrate and broaden its melting temperature range.^14^ More recently, our group developed a method to encapsulate pure salt hydrate PCM using nonaqueous Pickering emulsions as templates.^31^ In this system, alkylated graphene oxide (GO) nanosheets stabilized PCM-in-toluene emulsions and polymer was precipitated onto the surface of the droplets, forming a composite shell. Notably, the nanosheets served as nonspecific nucleating agents for the encapsulated magnesium nitrate hexahydrate (MNH), suppressing undercooling.

Utilization of additive manufacturing methods can substantially streamline fabrication processes, resulting in composites that are both cohesive and seamlessly integrated, while facilitating integration of TES systems into energy-efficient buildings. Indeed, additive manufacturing presents a significant opportunity for tailoring structures, including architecting highly compact heat exchangers with intricate fins or flow geometries, which are otherwise unattainable through traditional manufacturing.^32,33^ Geometry-enabled advancements increase heat transfer surface areas, thereby reducing heat transfer length scales.^34^ Printing allows for alternative materials to be evaluated, including composite polymers infused with additives for thermal conductivity enhancement.^33^ Further, lightweight, noncorrosive, and economical printed structures can be accessed that also require lower processing temperatures.^35,36^ To date, additive manufacturing has been used to effectively implement PCM-based Trombe walls through innovative designs,^37^ though organic PCMs have been predominantly used for 3D printing applications.^38−41^ This can likely be attributed to the fact that salt hydrates can dehydrate at the elevated temperatures required for fused deposition modeling (FDM), and they are incompatible with photocurable resins that are suitable for stereolithography (SLA). Previously, our group has reported the direct ink writing (DIW) of organic PCMs, specifically paraffins, within a matrix of a photocurable resin.^38^ The cured and printed structures exhibited an enthalpy of 103 J·g^–1^, derived exclusively from the 63 wt % of paraffin, with no contribution from the resin.

**Figure 1:**
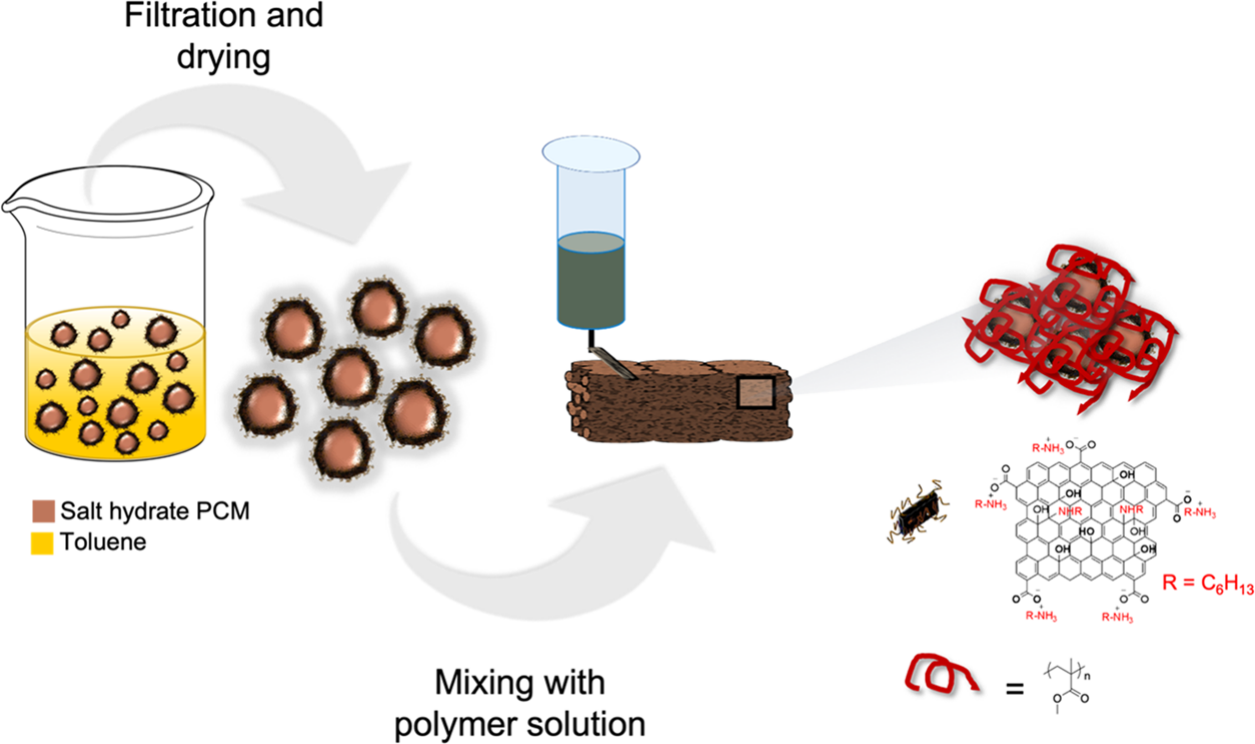
Schematic illustrating the formulation of salt-hydrate-containing inks and direct ink write printing of salt hydrate phase change materials (PCMs). Salt hydrate particles coated with alkylated graphene oxide (GO) nanosheets are mixed with toluene dispersions in polymer, extruded, and the printed object cured by evaporation of toluene.

Herein, we report a novel approach for printing composite hierarchical structures loaded with salt hydrate PCMs using a DIW additive manufacturing technique. DIW facilitates the customization of ink compositions by tailoring appropriate flow behavior, enabling the integration of functional components.^42^ We formulate inks by using alkylated GO-coated salt hydrate particles as rheological modifiers in toluene solutions of poly(methyl methacrylate) (PMMA). Inks with appropriate rheology can be printed at room temperature and then cured by solvent evaporation under ambient conditions. A schematic of this approach is shown in Figure 1. Remarkedly, this technique eliminates the need for prior encapsulation of the PCM. Two salt hydrates, magnesium nitrate hexahydrate (MNH, *T*_m_ = 89 °C) and zinc nitrate hexahydrate (ZNH, *T*_m_ = 36 °C), were employed to impart desirable thermophysical properties to the printed structures. The printed composites contained up to 70 wt % of the salt hydrate and underwent multiple thermal cycles without compromise to structural integrity, making full use of the encapsulated PCM’s melting and solidification. Further, we establish that the addition of carbon black to the ink enhances thermal conductivity by at least 33%. This straightforward approach provides monolithic composites containing salt hydrate PCMs, synergizing ink development and DIW to access desired thermal energy storage properties.

## Results and Discussion

Functional inks suitable for DIW were formulated by integrating salt hydrate particles coated with alkylated GO nanosheets (referred to as C_6_-GO nanosheets^43,44^) into dispersions of PMMA in toluene (see the SI for full details). Briefly, molten magnesium nitrate hexahydrate (MNH) was emulsified with toluene at 100 °C in the presence of C_6_-GO nanosheets. The emulsion was cooled to ambient temperature and the solidified MNH droplets coated in C_6_-GO were isolated and dried under reduced pressure to yield a brown powder of MNH particles. Figure S1A shows an optical microscopy image of the MNH-in-toluene emulsion, stabilized by C_6_-GO. Optical microscopy and SEM imaging of the dried MNH particles revealed nearly spherical particles 15–63 μm in diameter (Figures S1B and S1C, respectively). The average diameter of the particles was determined to be 37 μm using laser scattering particle size analysis (Figure S1D), in line with the size of the precursor emulsion droplets.

**Figure 2:**
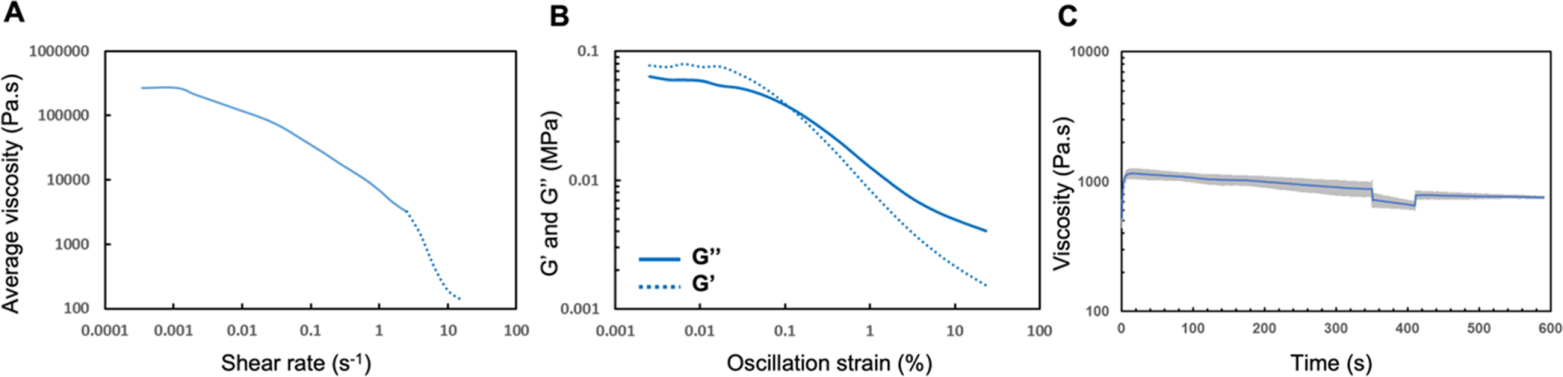
Characterization of the MNH-P ink composed of 6:10 (w/v) PMMA/toluene solution at a weight ratio of 1 g particles: 1 g solution. (A) Average viscosity of the ink as a function of shear rate; (B) storage modulus (*G*′, dotted line) and loss modulus (*G*″, solid line) as a function of oscillation strain; (C) three-interval thixotropy test with error bars represented in gray.

The solid MNH particles were mixed with a 6:10 (w/v) PMMA/toluene solution at a weight ratio of 1 g particles: 1 g solution, producing a viscous MNH-P ink as shown in Figure S2. The DIW-printability of this ink was confirmed by its rheological behavior, specifically that it is shear-thinning and thixotropic.^38^ Inks suitable for DIW should have the ability to be easily extruded from the print nozzle, then undergo a rapid restoration of original viscosity, and maintain its shape after curing.^45^Figure 2A shows that the average viscosity of the MNH-P ink decreased as the applied shear rate is increased from 0.0001 to 1000 s^–1^, thus exhibiting non-Newtonian, shear-thinning behavior. The PMMA solution itself was a Newtonian fluid (with viscosity independent of shear rate). Of note, the data in Figure 2A was truncated at high shear rates where MNH-P was expelled from the sides of the parallel plates of the rheometer, consistent with the behavior of thixotropic fluids. The yielding behavior of the MNH-P ink was evaluated by performing oscillatory strain sweeps. Figure 2B shows the storage (*G*′) and loss (*G*″) moduli of the MNH-P ink, as plotted against oscillation strain from 0.001 to 100%. The MNH-P ink exhibited viscoelastic behavior (where the elastic characteristics are more pronounced at low strains and viscous properties dominate at high strains), as well as exhibiting a yield point (identified as the crossover point of *G*′ and *G*”). This contrasts with the behavior of the PMMA solution that flowed at all levels of strain tested and did not display a yield point (Figure S4D). We evaluated several ink compositions, with different ratios of MNH particles and PMMA solutions, drawing upon our prior work on DIW of organic PCMs. Among these, the MNH-P ink demonstrated the highest loading of salt hydrate with appropriate rheological properties required for DIW. The favorable rheological properties of the ink can be attributed to interactions between MNH particles; shear-thinning and yielding behaviors were caused by disruption of interparticle interactions when the applied shear exceeded the yield stress, causing the ink to flow.^46^ Upon removal of the applied shear, the interactions were restored, enabling the extruded layers to self-support and recuperate to their properties. To confirm the thixotropy of the MNH-P ink, a three-interval thixotropy test (3ITT) was employed, as shown in Figure 2C. The sample was first held at a steady shear rate of 0.5 s^–1^ to achieve a viscosity plateau (first stage), after which the shear rate was increased to 1 s^–1^ for 60 s (second stage). Finally, the shear rate was returned to 0.5 s^–1^ and held for another 180 s (third stage). The MNH-P ink recovered 90% of its original viscosity after 8.1 s of the high shear rate.

As the desirable thixotropic properties of the MNH-P ink are attributed to particle characteristics (e.g., particle size and shape) and interparticle interactions, we posited that particles of other salt hydrates could be used to formulate inks for DIW, and in doing so, access thermal energy management across a different temperature range.^47,48^ Thus, ZNH particles were used as rheology modifiers in PMMA solutions to produce ZNH-P inks, in place of the MNH particles. ZNH particles were obtained via emulsification of molten ZNH in toluene at 55 °C in the presence of C_6_-GO nanosheets. Figure S3A shows an optical microscopy image of the emulsion, and Figures S3B,C show optical microscopy and SEM images, respectively, of the isolated C_6_-GO-coated ZNH particles. These ZNH particles were 19–78 μm in diameter, with an average diameter of 45 μm, as determined by laser scattering particle size analysis (Figure S3D). As with MNH particles, mixing ZNH particles with toluene solutions of PMMA imparts non-Newtonian, shear-thinning behavior to the ZNH-P ink as observed in its viscosity test (Figure S4A). The ZNH-P ink’s viscoelastic characteristics and yield point are evident in an oscillatory strain sweep (Figure S4B), and the thixotropic behavior confirmed with 3ITT, with the ink recovering 90% of its original viscosity after only 1.2 s (Figure S4C).

**Figure 3:**
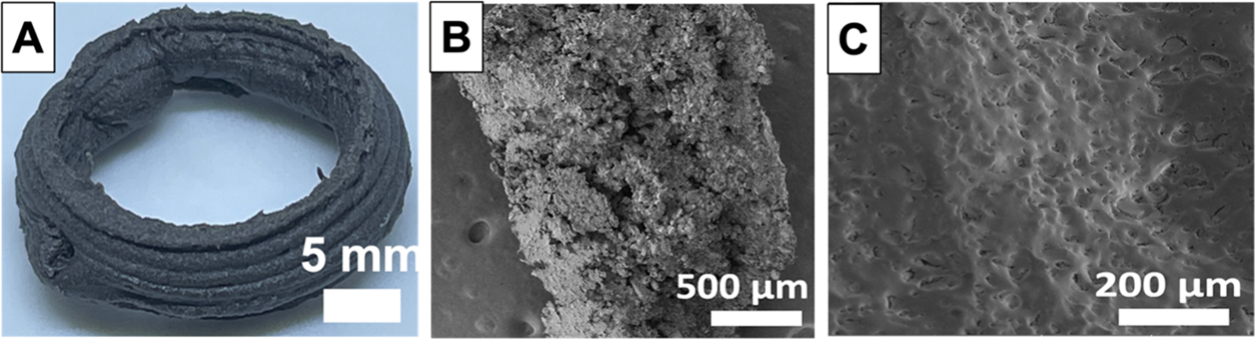
Characterization of the printed and cured object from MNH-P ink: (A) Digital image of the structure; (B) SEM image of the cross section of a filament; (C) SEM image of the surface of a filament.

After establishing ink formulations for DIW that exhibited optimal thixotropic properties without phase separation of the particles, the MNH-P and ZNH-P inks were successfully 3D printed using a Hyrel 3D Engine SR. To ensure curing of each printed layer, toluene was allowed to evaporate for a fixed time interval of 30 s between extrusion of consecutive layers. Digital images of printed and cured MNH-P and ZNH-P composites with a fixed layer height of 0.8 mm are shown in Figures 3A and S5A, respectively. The design of a hollow cylindrical structure in Figure 3A with the printed filaments closely stacked upon each other illustrates the potential of this technique for 3D printing polymeric heat exchangers components; the large surface areas and thin walls could facilitate effective heat transfer between the heat exchanger fluid and the salt hydrate PCM. Further, since salt hydrate is encased by the polymer, resistance to corrosion is expected.

The composition of the printed composites was evaluated with fourier-transform infrared spectroscopy (FTIR) (Figure S6), and the structure was characterized by scanning electron microscopy (SEM). The FTIR spectrum of the printed MNH-P composite shown in Figure S6A confirms the presence of MNH, as well as PMMA. This is supported by the peaks observed at 3390 cm^–1^ for O–H stretching, and 1630 and 1350 cm^–1^ corresponding to N–O stretching, characteristic of bulk MNH. Similar peaks are also observed for ZNH-P printed composite (Figure S6B), validating the presence of ZNH. Additionally, the FTIR spectra of both printed MNH-P and ZNH-P composites contain a stretching frequency at ∼3000 cm^–1^ which is assigned to C-H stretching originating from PMMA and C_6_-GO nanosheets. SEM micrographs of the cross sections of printed MNH-P and ZNH-P are presented in Figures 3B and S5B, respectively. These images reveal the presence of compacted regions consisting of salt hydrate particles embedded within the polymer matrix, along with small voids, which may be attributed to the DIW printing process (e.g., air bubbles). Figures 3C and S5C present SEM images that showcase the relatively smooth surfaces of MNH-P and ZNH-P printed composites containing a few micron-sized pores resulting from solvent evaporation during the curing process. SEM images support that the salt hydrate particles are enclosed in the polymer, without significant phase separation.

**Figure 4:**
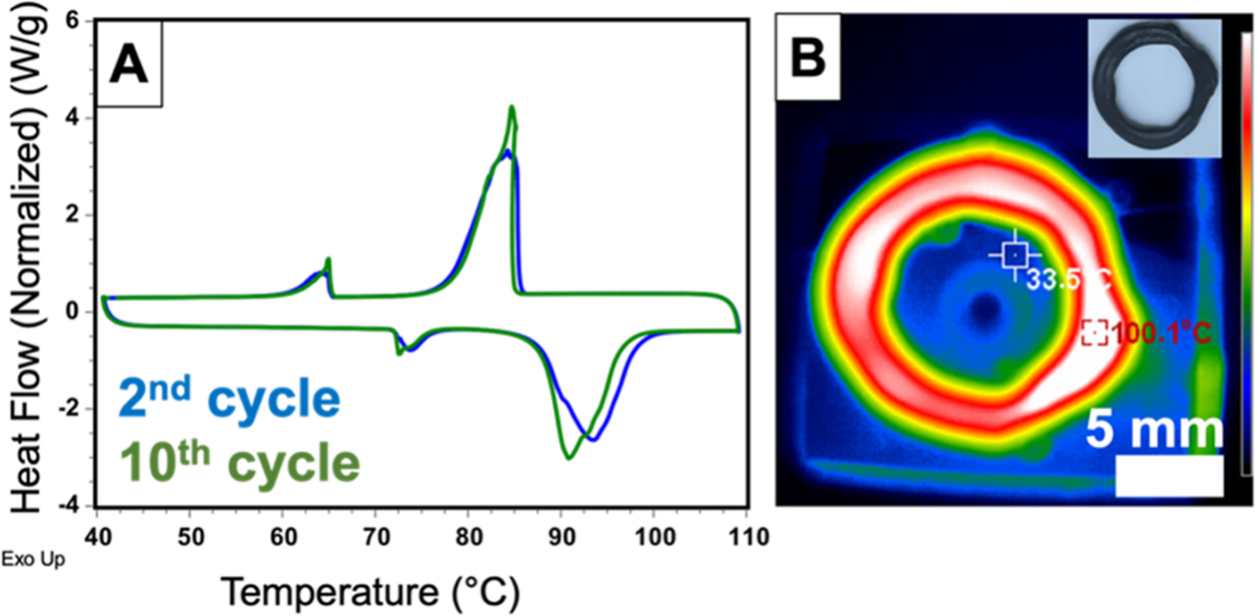
Thermal characterization of the object printed from the MNH-P ink: (A) DSC thermograms for the 2^nd^ and 10^th^ heating and cooling cycles. (B) Infrared thermal image of the object during heating, with the inset showing a digital image of the same object after cooling to ambient temperature.

The impact of the composite structure on the thermal performance of the printed MNH-P and ZNH-P composites was established using differential scanning calorimetry (DSC) and thermogravimetric analysis (TGA). Figure 4A shows the DSC thermograms of the printed MNH-P composite with an onset of melting at 88 °C, identical to that of bulk MNH (Figure S7A), denoting that the composition of the salt hydrate is conserved within the printed structure. Moreover, the printed MNH-P composite showed negligible undercooling with solidification at 88 °C; this contrasts with bulk MNH which shows an undercooling of ∼13 °C. This can be attributed to the surface-initiated solidification of MNH within the composite due to the presence of C_6_-GO nanosheets which acted as a nonspecific nucleating agent, as we previously reported.^31^ Notably, the solidification behavior of bulk MNH is consistent with the behavior of metastable liquids, which means that bulk MNH exhibits rapid exothermic solidification that overwhelms the constant cooling rate imposed within the DSC pan, thus resulting in a rapid transient temperature rise.^31^ Due to this, a loop is observed during the solidification exotherm of the bulk MNH (Figure S7A). The smaller endothermic and exothermic peaks observed at 71 and 65 °C, respectively, are common to salt hydrates and are associated with solid-solid phase transitions, as previously reported.^15^ The MNH-P printed composite is expected to contain ∼70 wt % of MNH based on ink design, and this is consistent with its latent heat value of 99.4 J·g^–1^ reflected during the 2^nd^ cycle of melting, in comparison with 140.2 J·g^–1^ of bulk MNH (i.e., all salt hydrate contributes to the thermal energy transition but polymer does not). The broad exothermic peaks for solidification in the MNH-P printed composite are due to steady solidification occurring in pockets of MNH, encased in polymer. Following 10 cycles of heating and cooling (shown in Figure S7B), the printed MNH-P composite maintained a latent heat of 98.4 J·g^–1^, demonstrating its robustness to repeated solid–liquid phase transitions in this closed system. To decouple the printing process from the ink composition, we cast a composite by mixing the same ratio of particles and PMMA solution then cured it through solvent evaporation under reduced pressure; the cast composite has the same composition as the MNH-P printed composite (∼70 wt % of MNH). The DSC thermogram for this as-cast system revealed an onset of melting and the latent heat value of 88 °C and 102 J·g^–1^, respectively (Figure S8), in line with the printed MNH-P composite (88 °C and 99.4 J·g^–1^, respectively). These data suggest that the printing process does not dramatically impact the properties of composites and that thermal performance is primarily dictated by the ink composition.

Similarly, DSC thermal transition profiles were obtained for bulk ZNH and printed ZNH-P composites. During its 2^nd^ cycle of melting, bulk ZNH had an onset of melting at 34 °C with an enthalpy of 147.2 J·g^–1^ (Figure S7C), whereas the ZNH-P printed composite containing ∼70 wt % of the salt hydrate showed an enthalpy of 98.8 J·g^–1^ with 33 °C as its onset of melting (3 heating/cooling cycles can be seen for the ZNH-P printed composite in Figure S7D). The solidification behavior of both bulk ZNH and the ZNH-P printed composite is similar to that of bulk MNH and MNH-P printed composite, respectively. Bulk ZNH exhibits a loop-like solidification exotherm whereas a broad exothermic peak is observed for the ZNH-P printed composite due to the encapsulated ZNH within the PMMA matrix. Notably, C_6_-GO nanosheets are not efficient for the nucleation of ZNH compared to their effectiveness as nucleating agents for MNH. While the composite printed with the ZNH-P ink portrayed good phase change cyclability, future work should address including nucleating agents in the ink formulation to minimize undercooling.

The resilience of the printed composites to thermal degradation was tested by examining changes in their structural integrity before and after exposure to heat. The printed MNH-P composite was placed on a pre-heated hot plate at 100 °C; Figure 4B depicts an infrared thermal image of this printed MNH-P composite during heating, demonstrating that the composite maintained its bulk shape as the MNH absorbed thermal energy and completely melted. This composite was then allowed to cool to ambient temperature. Upon cooling, the composite retained its structure as shown in the inset of Figure 4B. Notably, extended heating of the composite did lead to some leakage of molten MNH, assumedly through the micron-sized pores on the surface, likely due to capillary action. The TGA weight loss profiles of the printed composites further substantiated that confining the salt hydrates within the PMMA matrix enhanced thermal stability compared to the bulk, particularly at temperatures below 200 °C, beyond the typical operating temperature range of these salt hydrate PCMs. Both the printed MNH-P and the ZNH-P composites lost ∼10 wt % of their mass below 100 °C, which is likely the loss of water, whereas bulk salt hydrates undergo a weight loss of 25–35 wt % under the same conditions (Figure S9). After heating to 600 °C, both bulk PCMs and their printed composites retained a residual mass (∼15 wt % for MNH-based samples and ∼25 wt % for ZNH-based samples) which is likely due to the formation of oxides (e.g., magnesium oxide from MNH and zinc oxide from ZNH, as previously reported).^49^

The feasibility of PCM composites for practical applications is dictated by the rate at which heat can be stored or released. The intrinsically low thermal conductivities of salt hydrate PCMs can be enhanced by inclusion of thermally conductive fillers, such as metallic nanoparticles,^50−53^ nano-silica,^54,55^ and carbon nanomaterials.^54,56−58^ To improve the heat transfer rates of our salt hydrate PCM printed composites, we introduced carbon black within the MNH-P inks for DIW (e.g., in place of some of the salt hydrate particles). Carbon black was chosen due to its widespread use in increasing the thermal conductivities of organic PCMs^59,60^ and polymers,^61−63^ which typically have poor thermal conductivities. We hypothesized that uniform dispersion of a small quantity of carbon black throughout the PMMA matrix would build a thermally conductive network. To this end, carbon black and MNH particles were added to the PMMA/toluene solution and thoroughly homogenized to generate a viscous MNH-P-CB ink (see the SI for details). A cylindrical disk, 20 mm in diameter, was prepared from this ink and the thermal conductivity was measured using a steady-state vacuum-insulated hot-plate apparatus (Figure S10, Table S1).^64^ The average thermal conductivity of the printed MNH-P-CB composite was 0.9 W·m^–1^·K^–1^ and that of the printed MNH-P inks (i.e., without carbon black) had an average thermal conductivity of 0.6 W·m^–1^·K^–1^. These data established that the addition of ∼5 wt % of carbon black increased thermal conductivity by 33%.

**Figure 5:**
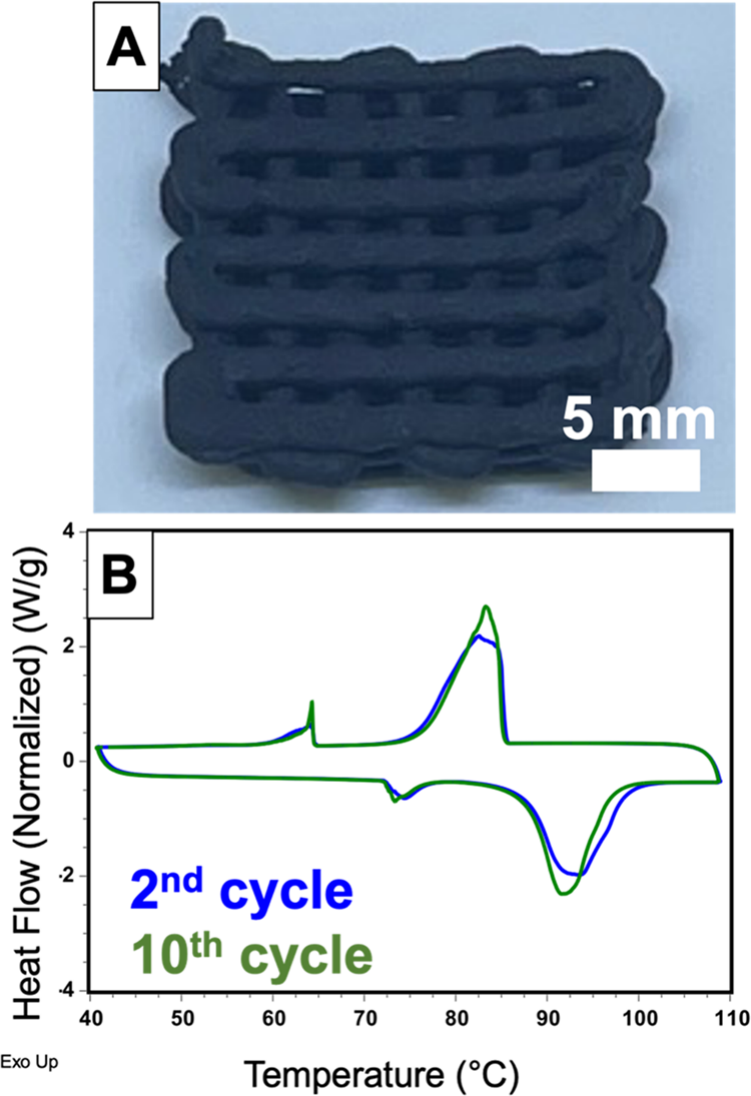
Characterization of printed MNH-P-CB composite. (A) Digital image of a cubic lattice; (B) DSC thermogram of a printed filament.

We then demonstrated the printability of the MNH-P-CB ink by DIW, as it possessed similar thixotropic and shear-thinning properties as the MNH-P ink. The MNH-P-CB ink was printed into a cubic lattice, as shown in Figure 5A, and its thermal phase transitions were characterized using DSC. As shown in Figure 5B, the 2^nd^ heating/cooling trace has an onset of melting at 88 °C, similar to the printed MNH-P composite discussed above, suggesting preservation of the MNH composition. This sample also exhibited a latent heat of 77.7 J·g^–1^ during its 2^nd^ heating cycle, consistent with the amount of MNH contained within the ink. The thermal cyclability of this composite is supported by retention of a latent heat of 77.6 J·g^–1^ after 10 heating/cooling cycles (Figure S11 shows all 10 heating/cooling cycles). Further, the printed lattice was characterized by surface and cross-sectional SEM imaging both before and after heating (Figure S12). These SEM images indicate no significant changes upon cycling, though the surface of the MNH-P-CB appears slightly smoother after thermal cycling. The chemical composition of MNH-P-CB was evaluated by elemental analysis using energy-dispersive X-ray spectroscopy (EDS) coupled with SEM, again before and after thermal cycling (Figure S13). Magnesium was detected in both the samples, confirming the presence of MNH, yet signal intensity is not consistent with the composition determined by latent heat values, likely due to the domination from carbon near the surface (carbon black, PMMA, and C_6_-GO).

## Conclusions

In summary, we report a facile formulation and 3D printing of inks composed of PCM particles and polymer by leveraging salt hydrate particles as rheology modifiers and DIW printing. Microscopic analysis of the printed and cured structures confirmed no phase separation and that the salt hydrate particles were dispersed through the PMMA matrix, even at salt hydrate content of up to 70 wt %. Thermal analyses validated the preservation of the salt hydrate composition and the reduction in undercooling in these printed polymeric composites. Moreover, these composites exhibit good thermal stability over at least 10 heating/cooling cycles without significant macroscopic changes in their structure. Notably, this approach eliminates the need for microencapsulation of salt hydrates prior to integration into the polymer composite. It further facilitates the incorporation of fillers such as carbon black, which resulted in a 33% enhancement in thermal conductivity. We demonstrate that this approach is adaptable to particles of different salt hydrates, and thus can be used to tailor the temperature at which thermal energy management can be performed. Our ongoing research focuses on expanding the range of matrix materials for DIW to optimize the stability and permeability of the composites. For instance, the use of non-solvent-based resins can reduce the formation of pores on the surface of the printed composites caused by solvent evaporation during the curing process. Overall, this new approach to formulate inks for 3D printing holds significant promise for manufacturing PCM composites with tailored properties, thus highlighting the potential of adopting salt hydrates in diverse thermal energy storage applications.

## Supporting Information Available

Materials and methods; optical microscopy image of MNH-in-toluene emulsion stabilized by C_6_-GO nanosheets, optical microscopy image of dried MNH particles, SEM image of dried MNH particles, particle size distribution of MNH particles (Figure S1); schematic illustrating formulation of MNH-P ink (Figure S2); optical microscopy image of ZNH-in-toluene emulsion stabilized by C_6_-GO nanosheets; optical microscopy image of dried ZNH particles; SEM image of dried ZNH particles; particle size distribution of ZNH particles (Figure S3); average viscosity of ZNH-P ink as a function of shear rate, storage modulus (*G*′, dotted line) and loss modulus (*G*″, solid line) of ZNH-P ink as a function of oscillation strain, three-interval thixotropy test for ZNH-P ink with error bars represented in gray, storage modulus (*G*″, dotted line) and loss modulus (*G*″, solid line) of the polymer solution (PMMA dispersed in toluene) as a function of oscillation strain (Figure S4); digital image of letter “S” printed using ZNH-P ink, SEM image of the cross section of ZNH-P printed composite, SEM image of the surface of ZNH-P printed composite (Figure S5); offset FTIR spectra for bulk MNH (blue trace), MNH particles coated with alkylated GO nanosheets (orange trace), and MNH-P printed composite (gray trace), bulk ZNH (blue trace), ZNH particles coated with alkylated GO nanosheets (orange trace), and ZNH-P printed composite (gray trace) (Figure S6); DSC profiles for bulk MNH (second cycle), MNH-P printed composite (10 cycles), bulk ZNH (second cycle), ZNH-P printed composite (three cycles) (Figure S7); DSC profile for the cast composite with ink composition identical to MNH-P (Figure S8); TGA weight loss profiles for printed composite using MNH-P ink (solid line) and bulk MNH (dotted line), printed composite using ZNH-P ink (solid line) and bulk ZNH (dotted line) (Figure S9); digital images of steady-state setup used for thermal conductivity measurements, representative sample used to measure thermal conductivity (Figure S10); DSC thermogram of the MNH-P-CB-printed composite (Figure S11); SEM images of surface of MNH-P-CB-printed composite prior to heating, cross section of MNH-P-CB-printed composite prior to heating, surface of MNH-P-CB-printed composite following 10 heating/cooling cycles, cross section of MNH-P-CB-printed composite following 10 heating/cooling cycles (Figure S12); SEM-EDS analysis of MNH-P-CB-printed composite before heating and after heating (Figure S13); and representative position and temperature readings of all of the thermocouples, including the extrapolated junction temperatures (Table S1) (PDF)
