# Supplementary material for: Printing Composites with Salt Hydrate Phase Change Materials for Thermal Energy Storage
Source: ACS Appl Eng Mater. 2023 Aug 4;1(8):2279–87. doi: 10.1021/acsaenm.3c00324 (PMC10862487; doi:10.1021/acsaenm.3c00324)
Supplement: Supplementary file 1 — em3c00324_si_001.pdf [file em3c00324_si_001.pdf]

Supporting Information

Printing Composites with Salt Hydrate Phase Change Materials for Thermal Energy Storage

*Sarah N. Lak,[1] Chia-Min Hsieh,[1] Luma AlMahbobi,[2] Yifei Wang,[2] Anirban Chakraborty,[3] Choongho Yu,[3] Emily B. Pentzer[1,2]**

[1]Department of Chemistry, Texas A&M University, College Station, TX 77843, USA
[2]Department of Materials Science and Engineering, Texas A&M University, College Station, TX 77843, USA
[3]Department of Mechanical Engineering, Texas A&M University, College Station, TX 77843, USA
*emilypentzer@tamu.edu

Materials: Graphite, sulfuric acid (H_2SO_4, 95.0-98.0%), and potassium permanganate ($KMNO_4$, ≥99.0%) were purchased from Sigma Aldrich. Hydrogen peroxide (H_2O_2, 30%), isopropanol (≥99.5%), toluene (≥99.5%), *N,N*-Dimethylformamide (DMF, ≥99.8%), hexylamine (99%), methanol (99.8%), hexanes (≥98.5%), carbon black (99.9+%, S.A. 75 m^2/g, bulk density 170-230 g/L), magnesium nitrate hexahydrate (MNH, 98.0-102.0%) and zinc nitrate hexahydrate (ZNH, 98%) were purchased from Fisher Scientific. Poly(methyl methacrylate) (PMMA) beads were purchased from Amazon with number-average molar mass (M_n) of 84.1 kDa and dispersity (*Đ*) of 2.58 as determined by size exclusion chromatography (SEC), calibrated relative to polystyrene standards. All reagents were used as received.

Instrumentation: Centrifugation was performed by using a Thermo Scientific Sorvall ST 8 centrifuge. Sonication was performed with a Fisherbrand CPX3800 Ultrasonic Bath 5.7 L. Vortexing was done with a Fisherbrand vortex mixer, model 9454FIALUS. A hand-held emulsifier (model number 985370-XL) from BioSpec Products, Inc. was used for shearing. A laboratory blender purchased from Waring Commercial (model 7010S) was used for blending dry nanosheets into a fine powder. The optical microscopy images were taken by an AmScope microscope purchased from Amscope with the camera (model A35180U3) purchased from Amazon. Digital images were taken with an iPhone 12 camera. Fourier Transform Infrared Spectroscopy (FTIR) data was collected using a JASCO FT/IR-4600 with a ZnSe/diamond prism with 16 scans in ATR mode. SEM images were collected on a TESCAN VEGA scanning electron microscope with an acceleration voltage of 2 kV. Samples for SEM were also sputter coated with 10 nm of Au. SEM-EDS analysis was performed on a TESCAN VEGA SEM equipped with an Oxford Instruments EDS system. Differential Scanning Calorimetry (DSC) data was collected using a TA Instruments DSC 2500. All DSC experiments were carried out in aluminum pans with hermetic seals. The ramp rates used for DSC experiments were as follows: 10 °C/min for heating and cooling of all MNH based samples and 10 °C/min for heating and 3 °C/min for cooling of all ZNH based samples. Thermogravimetric analysis (TGA) was performed on a TA Instruments TGA 5500. The procedure used for all TGA experiments was as follows: ramp 10 °C/min to 100 °C, isothermal 5 min, ramp 20 °C/min to 600 °C. All TGA runs were performed under nitrogen. Particle size

analysis was carried out using Horiba Partica LA- 960 particle sizer. Both MNH and ZNH particles were dispersed in toluene for size analysis using the fraction cell. Thermal images were recorded with a 320 × 240 IR resolution infrared camera (HT-A2, Hti). All inks were mixed in a Thinky AR-100 planetary mixer. 3D printing was performed on a Hyrel 3D Engine SR with an SDS-10 syringe extrusion head. The rheology of all inks was evaluated using a TA Instruments DHR-2 Rheometer with a 40 mm parallel plate.

Synthesis of C_6-GO nanosheets: C_6-GO nanosheets were prepared by alkylating GO nanosheets as previously reported.[1] GO nanosheets were first synthesized using a modified Hummers' method, washed, and dried under reduced pressure.1,2 Dry GO nanosheets (100 mg) were then dispersed in DMF (50 mL) using bath sonication for 30 minutes This GO dispersion in DMF was heated at 55 °C and hexylamine (2.5 mL) was added to it and the system heated to 55 °C for 24 hours. Synthesized C_6-GO nanosheets were then collected as a pellet by centrifugation, the supernatant was discarded, then the pellet was resuspended in a mixture of methanol and hexanes to remove any excess hexylamine, discarding the supernatant each time. Washed C_6-GO nanosheets were dried under reduced pressure for about 3 hours and blended into a fine powder using a laboratory blender.

Synthesis of MNH particles: Solid MNH (5.25 g) was heated in a sealed 250 mL round bottom flask at 95 °C, until all of it melted. C_6-GO was dispersed in toluene (70 mg nanosheets in 35 mL) by sonication to make a 2 mg/mL dispersion. This dispersion of C_6-GO nanosheets in toluene was added to the molten MNH and the system emulsified for 5 minutes using maximum speed of the hand-held emulsifier, while heating at 100 °C. The resulting emulsion was cooled at room temperature and toluene was then decanted. Residual solvent was removed by drying the alkylated nanosheet-coated MNH particles under reduced pressure for about 4 hours to obtain a solid, brown powder of MNH particles.

Synthesis of ZNH particles: Same procedure was observed as described for MNH particles, except solid ZNH (5 g) was heated at 55 °C and 25 mL of 2 mg/mL dispersion of C_6-GO nanosheets in toluene was added to molten ZNH. This biphasic system was emulsified for 5 minutes using maximum speed of the hand-held emulsifier, while heating at 55 °C.

Preparation of inks: In a 20 mL scintillation vial, PMMA beads (6 g) were heated in toluene (10 mL) at 50 °C overnight and then sonicated until a clear 6:10 (w:v) PMMA/toluene solution was obtained. MNH particles were added to this solution at a weight ratio of 1 g particles: 1 g solution. This mixture was then thoroughly homogenized by mixing in a Thinky AR-100 at 2000 rpm to produce MNH-P inks. Same procedure was observed to obtain ZNH-P inks, except ZNH particles were used instead of MNH particles. For MNH-P-CB inks, PMMA beads (5 g) were heated in toluene (10 mL) at 50 °C overnight and then sonicated until a clear 5:10 (w:v) PMMA/toluene solution was obtained. To 1 g of this solution, 900 mg of MNH particles and 70 mg carbon black was added and thoroughly homogenized by mixing in a Thinky AR-100 at 2000 rpm.

3D printing: Each ink was loaded into a 10 mL Luer-Lok syringe equipped with an 14G nozzle (1.600 ± 0.076 mm inner diameter) and the loaded syringe was then inserted into an extrusion cartridge attached to the 3D printer. During the printing process, each printed layer was cured by solvent evaporation by allowing 30 seconds between each layer. Other printing parameters used

are summarized as follows: nozzle temperature of 22 °C, layer height of 0.8 mm, infill density of 70% with rectilinear infill pattern, and infill speed, first layer speed, and travel speed of 20 mm/s.

Rheology experiments: The rheological properties of all inks used for DIW were evaluated using a TA Instruments DHR-2 Rheometer with a 40 mm parallel plate. All tests were conducted at 25°C with a gap distance of 500 μm using a rotational rheometer. The viscosity of each ink was measured at shear rates ranging from 0.001 to 1000 Hz. Additionally, a stress amplitude sweep was performed from 0.001 to 1000 Pa at a frequency of 1 Hz to determine the storage and loss moduli. The three-interval thixotropy test (3ITT) was performed by first keeping the samples at a shear rate of 0.5 Hz until a plateau on the viscosity-shear rate curves was observed (first stage). The shear rate was then adjusted to 1.0 Hz and held for 60 s (second stage). During the third stage, the shear rate was tuned back to 0.5 Hz and held for 180 s. All rheology experiments were performed with a cover to prevent the evaporation of the solvent in the sample. Each test was repeated twice for every sample to obtain average values.

Thermal conductivity measurements: Thermal conductivity values at 25 ± 1 °C were obtained using a steady-state vacuum-insulated hot-plate apparatus, comprising of two Al rods (20 mm in diameter) with a heater attached above the top rod and cooling water circulated below the bottom rod. Samples possessing same diameter as the Al rods (20 mm) were placed at the junction between the two rods. Eight T-type thermocouples attached at pre-determined positions recorded the temperature profiles across the length of the rods. Linear trendlines obtained by plotting the temperature readings of the thermocouples against their relative positions (one trendline for the top rod's thermocouples and another trendline for the bottom rod's thermocouples) were used to extrapolate the junction temperatures between: (1) top rod of the setup and the sample and (2) bottom rod of the setup and the sample, as shown in Table S1. The difference between the junction temperatures can be considered as the temperature drop across the thickness of the sample (ΔT_{drop}). This ΔT_{drop} is a measure of the thermal contact resistance of the sample, present at the junction between the two rods. The ΔT_{drop} at the junction, thickness of the sample and heat flow across the rods were used to compute the thermal conductivity of a sample. To reduce heat loss via convection, the measurement was carried out under vacuum (0.39 kPa absolute pressure). Validation of the thermal conductivity results obtained using our steady state vacuum-insulated setup has been previously reported.[3] Each sample was subjected to 5 rounds of measurement and the average values for thermal conductivity were obtained.

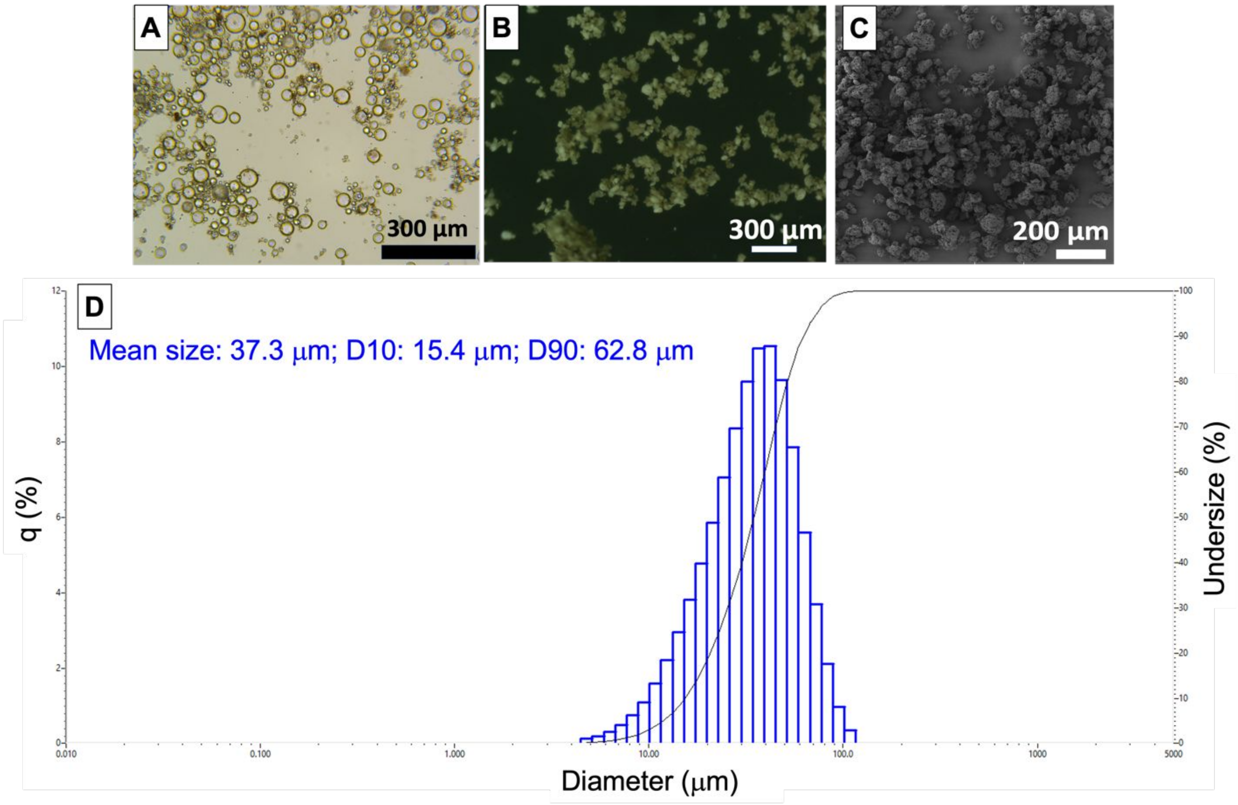

Figure S1. A) Optical microscopy image of MNH-in-toluene emulsion stabilized by C_6-GO nanosheets; B) Optical microscopy image of dried MNH particles; C) SEM image of dried MNH particles; D) Particle size distribution of MNH particles.

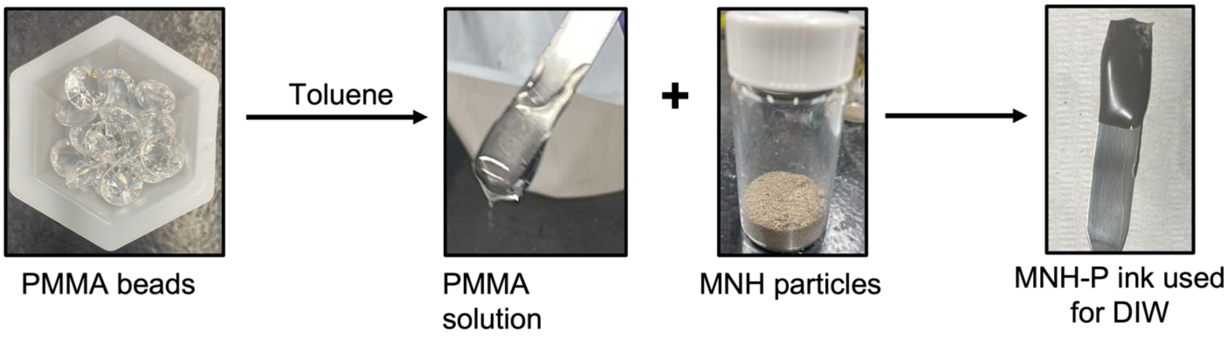

Figure S2. Schematic illustrating formulation of MNH-P ink.

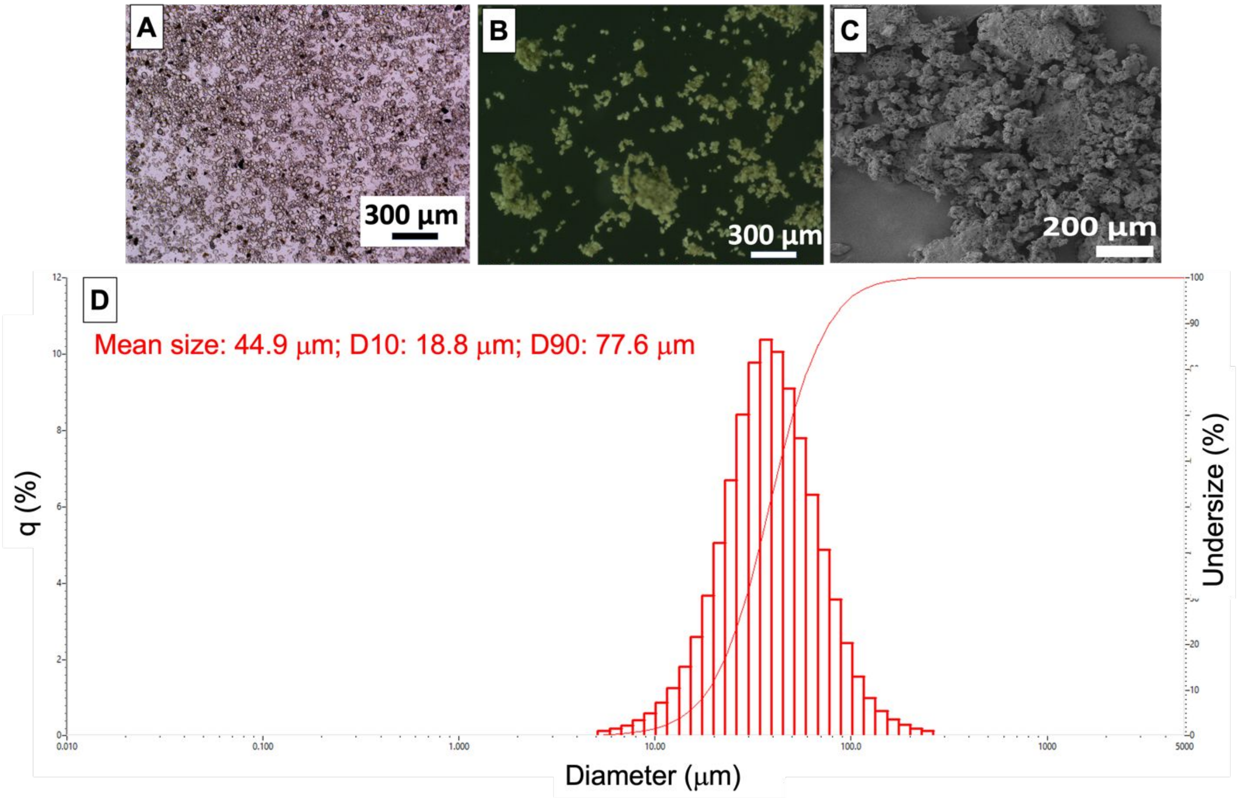

Figure S3. A) Optical microscopy image of ZNH-in-toluene emulsion stabilized by C_6-GO nanosheets; B) Optical microscopy image of dried ZNH particles; C) SEM image of dried ZNH particles; D) Particle size distribution of ZNH particles.

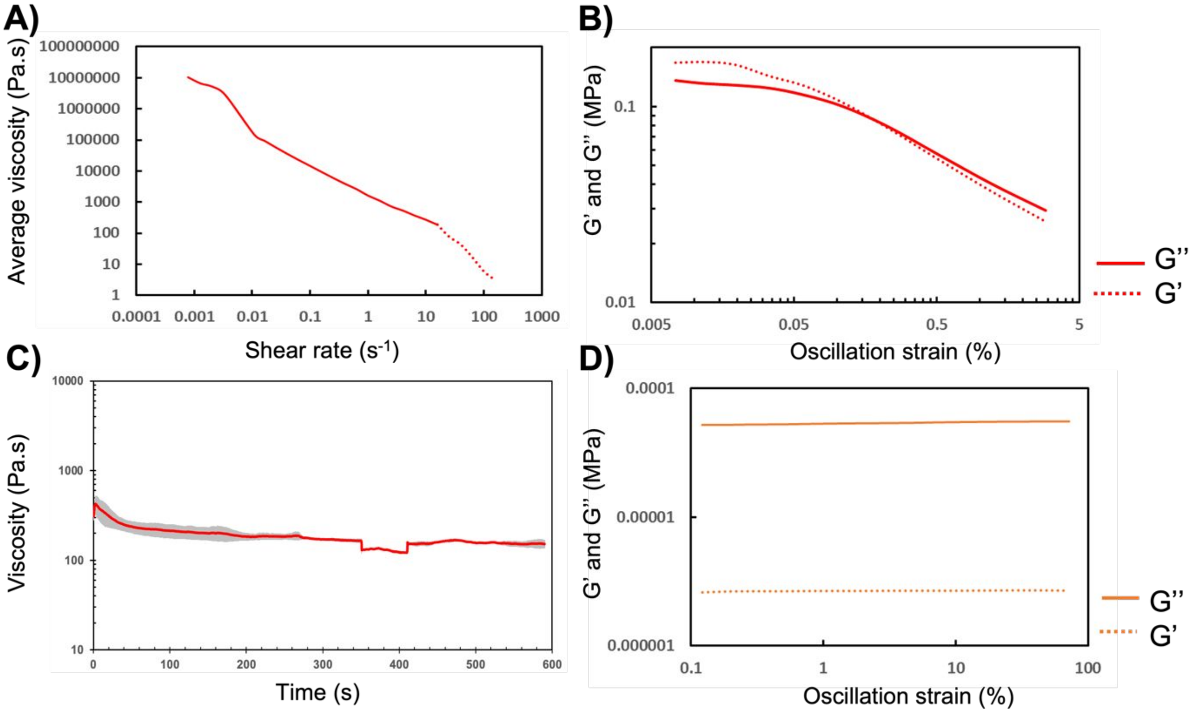

Figure S4. A) Average viscosity of ZNH-P ink as a function of shear rate; B) Storage modulus (G', dotted line) and loss modulus (G'', solid line) of ZNH-P ink as a function of oscillation strain; C) Three-interval thixotropy test for ZNH-P ink with error bars represented in gray; D)) Storage modulus (G', dotted line) and loss modulus (G'', solid line) of the polymer solution (PMMA dispersed in toluene) as a function of oscillation strain.

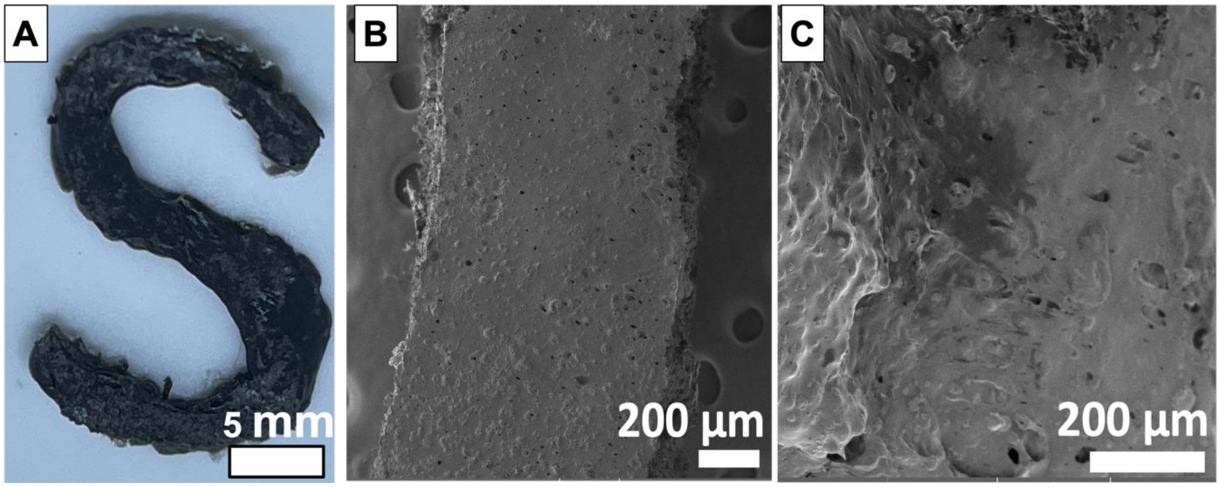

Figure S5. A) Digital image of letter 'S' printed using ZNH-P ink; B) SEM image of the cross-section of ZNH-P printed composite; C) SEM image of the surface of ZNH-P printed composite.

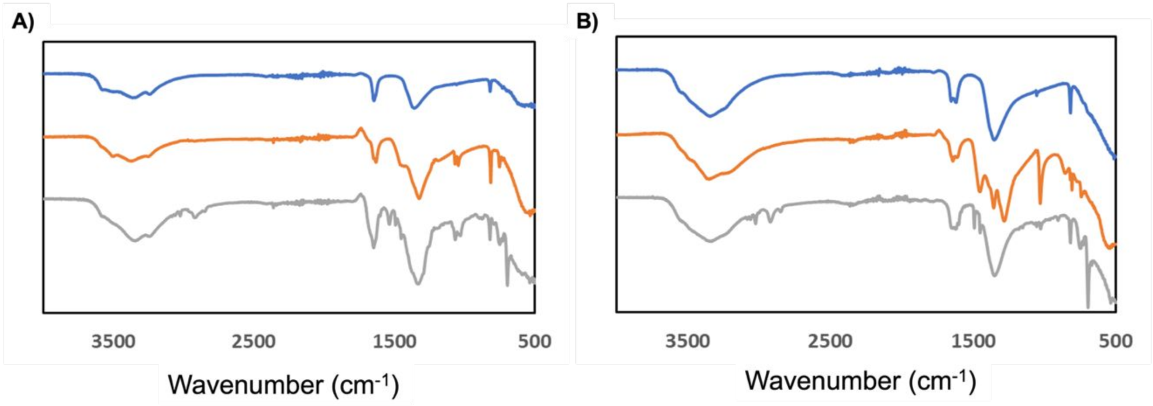

Figure S6. Offset FTIR spectra for: A) bulk MNH (blue trace), MNH particles coated with alkylated GO nanosheets (orange trace), and MNH-P printed composite (gray trace); B) bulk ZNH (blue trace), ZNH particles coated with alkylated GO nanosheets (orange trace), and ZNH-P printed composite (gray trace).

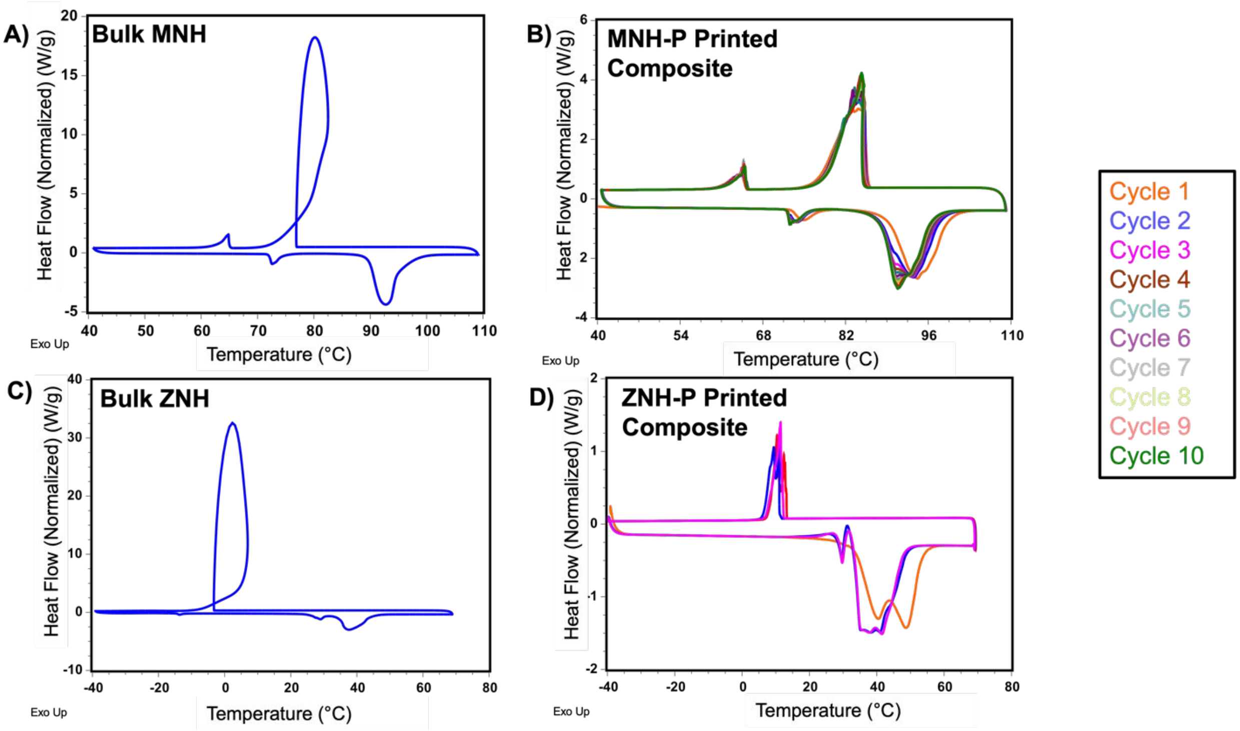

Figure S7. DSC profiles for: A) bulk MNH (second cycle); B) MNH-P printed composite (ten cycles); C) bulk ZNH (second cycle); D) ZNH-P printed composite (three cycles). Undercooling can be observed for both bulk MNH and bulk ZNH. Undercooling in salt hydrates indicates a nucleation-limited process where solidification occurs below the equilibrium melting temperature.[4]

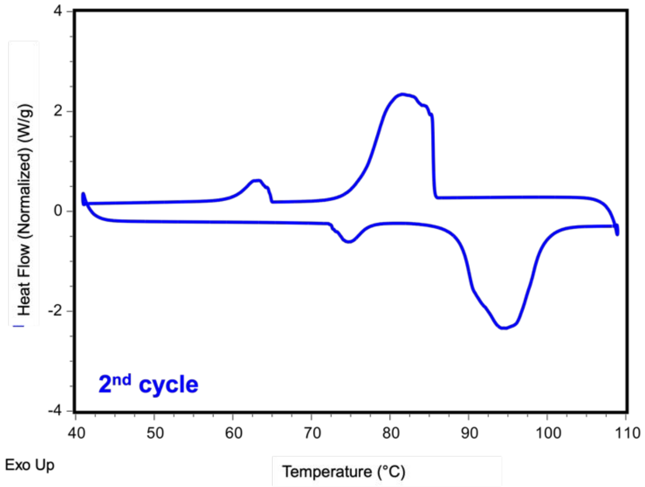

Figure S8. DSC profile for the cast composite with ink composition identical to MNH-P.

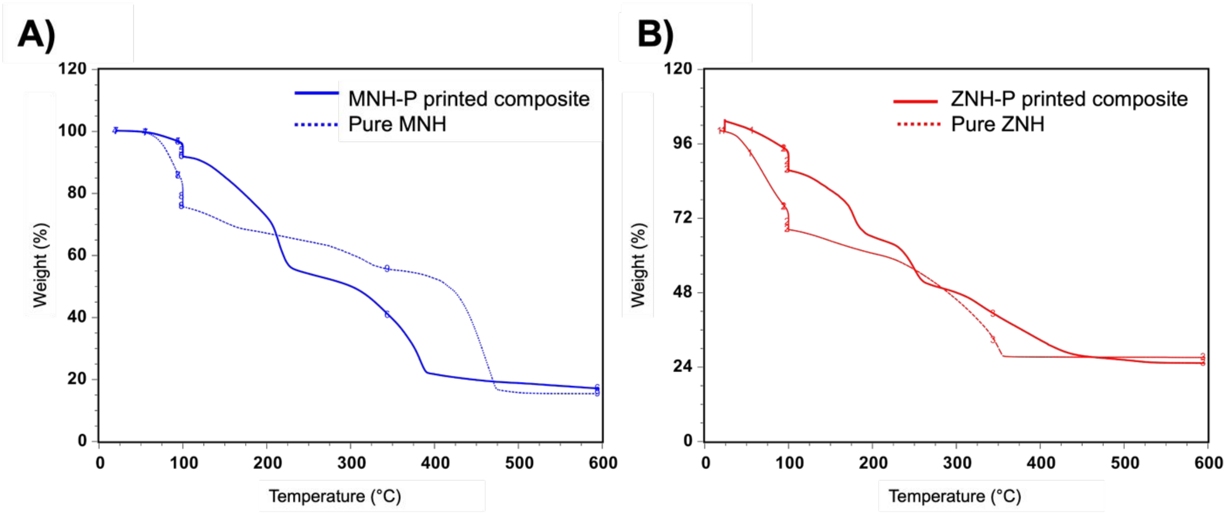

Figure S9. TGA weight loss profiles for: A) printed composite using MNH-P ink (solid line) and bulk MNH (dotted line); B) printed composite using ZNH-P ink (solid line) and bulk ZNH (dotted line).

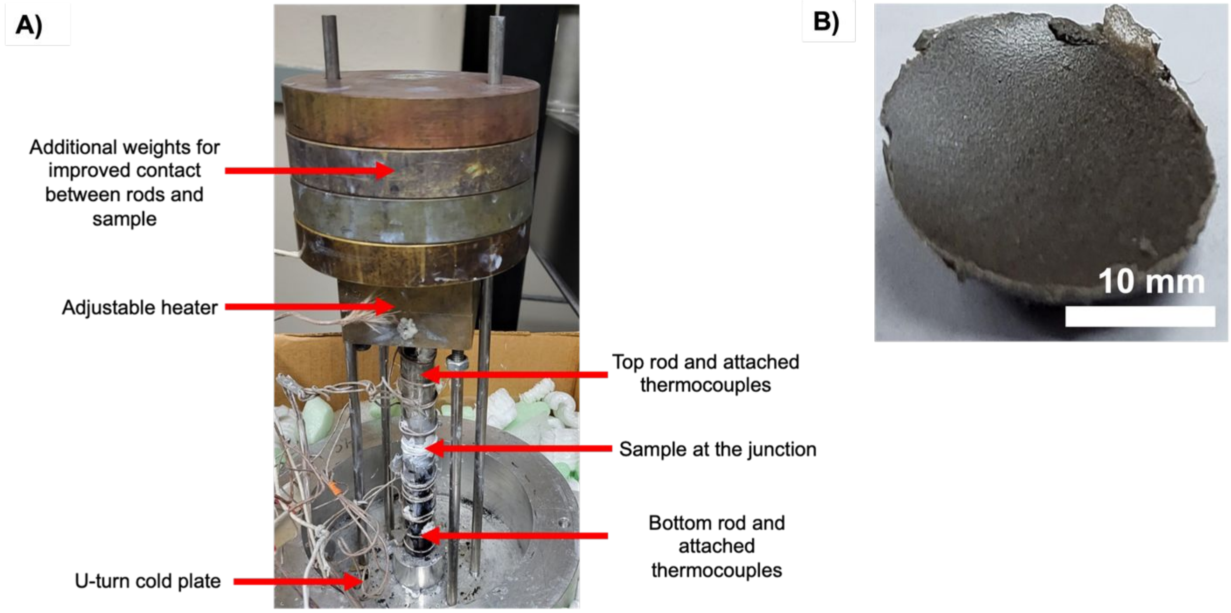

Figure S10. Digital images of: A) steady-state setup used for thermal conductivity measurements; B) representative sample used to measure thermal conductivity.

Table S1: Representative position and temperature readings of all the thermocouples, including the extrapolated junction temperatures.

Measurement	Distance (mm)	Temperature (°C)
Thermocouple 1 (top rod)	20	31.9
Thermocouple 2 (top rod)	35	30.5
Thermocouple 3 (top rod)	50	29.1
Thermocouple 4 (top rod)	65	27.7
Junction between top rod and sample	***80***	***26.3***
Junction between bottom rod and sample	***81.7***	***23.9***
Thermocouple 5 (bottom rod)	96.7	22.6
Thermocouple 6 (bottom rod)	111.7	21.3
Thermocouple 7 (bottom rod)	126.7	20.0
Thermocouple 8 (bottom rod)	141.7	18.6

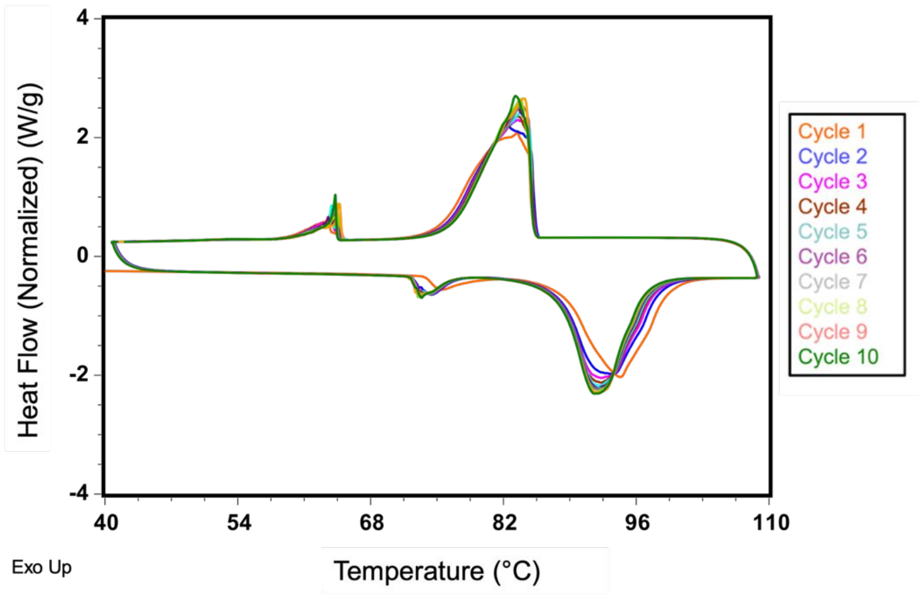

Figure S11. DSC thermogram of the MNH-P-CB printed composite.

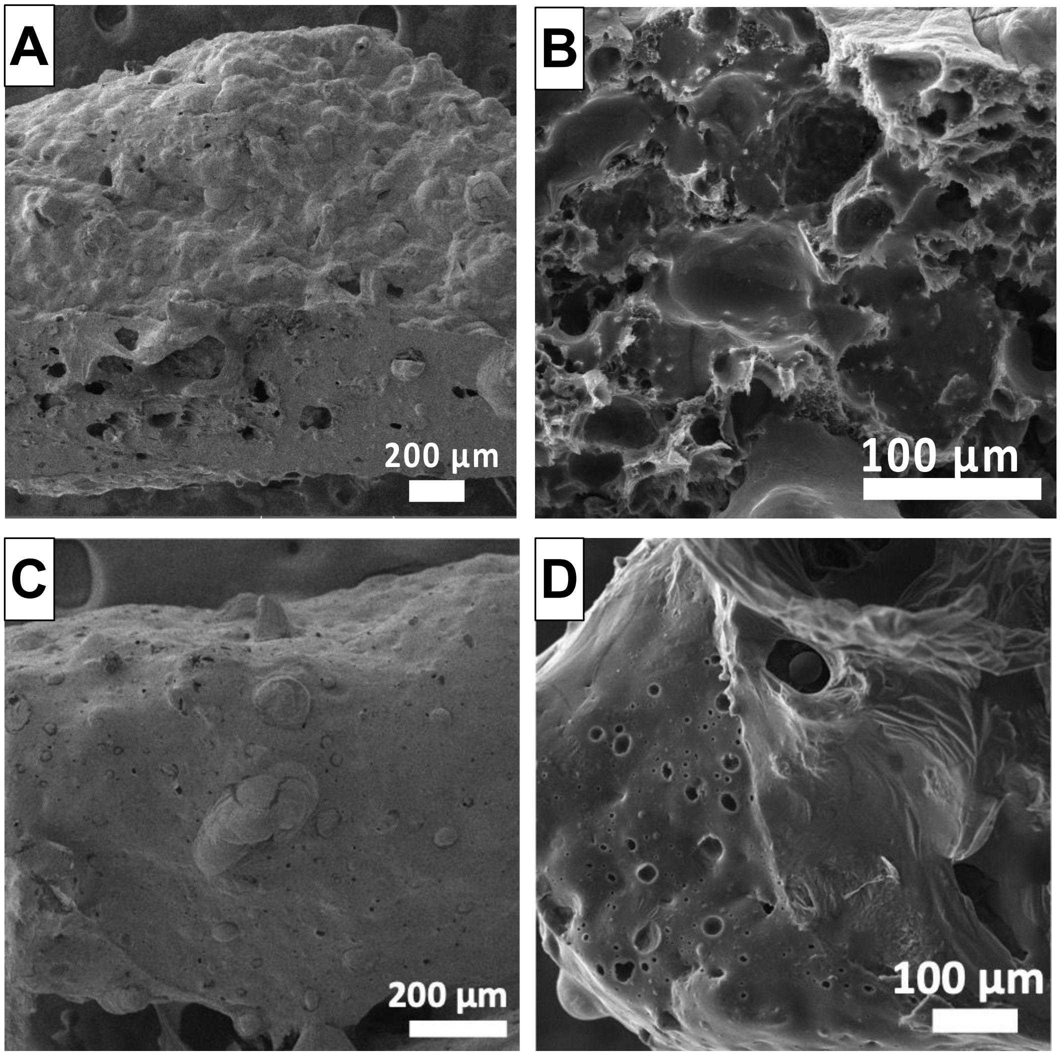

Figure S12. SEM images of: A) surface of MNH-P-CB printed composite prior to heating; B) cross-section of MNH-P-CB printed composite prior to heating; C) surface of MNH-P-CB printed composite following 10 heating/cooling cycles; D) cross-section of MNH-P-CB printed composite following 10 heating/cooling cycles.

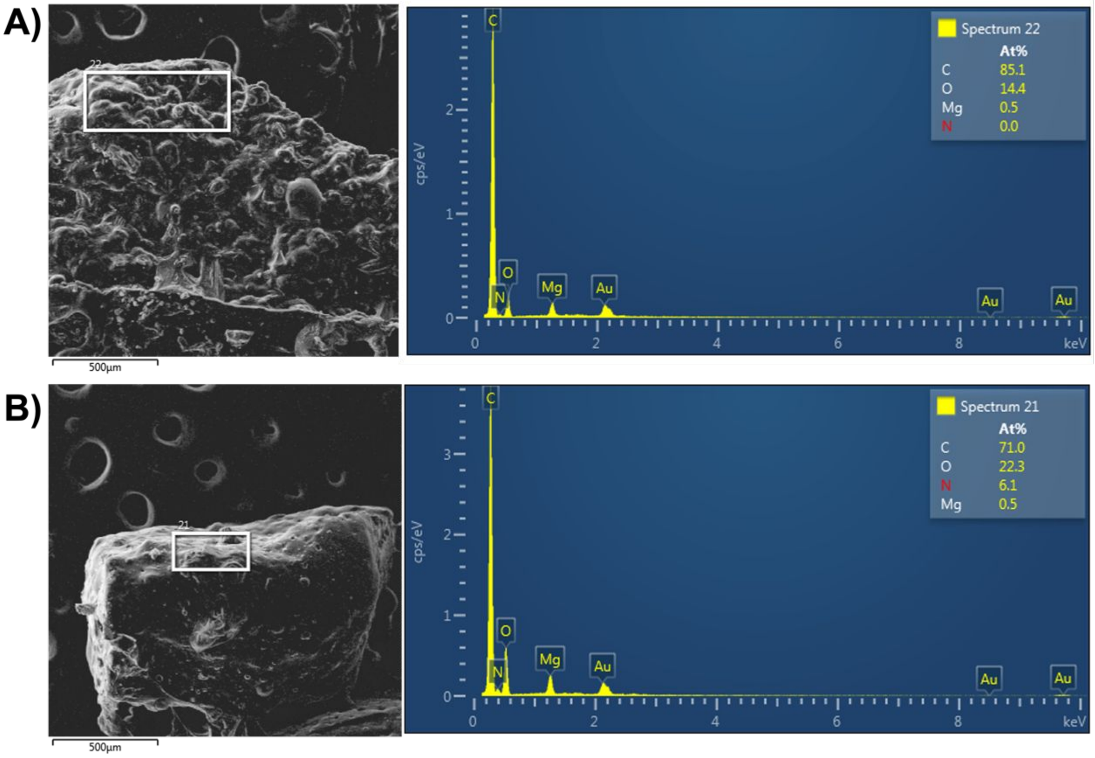

Figure S13. SEM-EDS analysis of MNH-P-CB printed composite: A) before heating; B) after heating.

References

(1) Rodier, B.; De Leon, A.; Hemmingsen, C.; Pentzer, E. Controlling Oil-in-Oil Pickering-Type Emulsions Using 2D Materials as Surfactant. *ACS Macro Lett* **2017**, *6* (11). https://doi.org/10.1021/acsmacrolett.7b00648.

(2) Luo, Q.; Wang, Y.; Yoo, E.; Wei, P.; Pentzer, E. Ionic Liquid-Containing Pickering Emulsions Stabilized by Graphene Oxide-Based Surfactants. *Langmuir* **2018**, *34* (34), 10114–10122. https://doi.org/10.1021/acs.langmuir.8b02011.

(3) Chakraborty, A.; Noh, J.; Mach, R.; Shamberger, P.; Yu, C. Thermal Energy Storage Composites with Preformed Expanded Graphite Matrix and Paraffin Wax for Long-Term Cycling Stability and Tailored Thermal Properties. *J Energy Storage* **2022**, *52*, 104856. https://doi.org/10.1016/j.est.2022.104856.

(4) Shamberger, P.; O'Malley, M. Heterogeneous Nucleation of Thermal Storage Material LiNO3·3H2O from Stable Lattice-Matched Nucleation Catalysts. *Acta Mater* **2015**, *84*. https://doi.org/10.1016/j.actamat.2014.10.051.
